# Improving the Food Environment in Hospitals and Senior Meal Programs

**DOI:** 10.5888/pcd15.170429

**Published:** 2018-02-15

**Authors:** Ann Lowenfels, Mary Jo Pattison, Anne M. Martin, Cindy Ferrari

**Affiliations:** 1 Bureau of Chronic Disease Evaluation and Research, New York State Department of Health, Albany, New York; 2 Bureau of Community Chronic Disease Prevention, New York State Department of Health, Albany, New York

## Abstract

Most Americans consume more than the recommended daily level of sodium, and tools are needed to assess and improve food practices related to sodium. We describe how the Sodium Practices Assessment Tool (SPAT) was developed and used in 19 hospitals and senior meal facilities in upstate New York. Initial results identified opportunities for improvement in food preparation, presentation, and purchasing practices to reduce sodium consumption. Pre–post comparison results showed significant increases in the use of herbs, spices, unsalted butter, fruits and vegetables, and in the availability of lower-sodium foods. Food service sites can use SPAT to assess sodium practices, inform development of action plans, and measure change over time.

## Sodium in the American Diet

Excessive sodium consumption contributes to high blood pressure, which is a major risk factor for heart disease and stroke (1,2). Sodium consumption has increased dramatically over the past 30 years (3), and most Americans now consume more than recommended daily levels (4). Americans consume an average of 3,375 mg of sodium per day, whereas dietary guidelines recommend consuming less than 2,300 mg (5,6). Reducing sodium consumption to recommended levels could eliminate 11 million cases of hypertension and save $10 billion to $24 billion in health care costs per year in the United States (7,8). One study showed that reducing sodium consumption by 1,200 mg per day would reduce annual health care costs for California, Texas, Florida, and New York State by more than $1 billion each (9).

Most of the sodium that Americans consume is added to food outside the home (10,11). Because sodium in the food supply is both widespread and often hidden, people cannot easily reduce their own consumption levels. The Institute of Medicine, the American Heart Association, and other expert panels recommend reducing sodium consumption by improving the overall food environment in the United States (12,2,13). The Centers for Disease Control and Prevention (CDC) has responded to this call by supporting programs that reduce sodium intake at the community level (14).

Food service programs that want to contribute to reductions in sodium intake need to assess their practices, implement appropriate changes, and monitor progress over time. Few tools exist to support these activities. We describe how a food service tool was developed and used in hospitals and for senior meal programs in New York State to assess sodium practices, inform development of action plans to reduce sodium intake, and measure change over time.

## Sodium Practices Assessment Tool

In 2013, New York State Department of Health (NYSDOH) received CDC funding to reduce sodium consumption in 5 upstate counties. Local health departments partnered with hospitals and senior meal programs in those counties to reduce the sodium content of meals served. The project team implemented varied strategies, including adopting food service guidelines and nutrition standards, reformulating menus and menu items, changing food preparation and presentation methods, modifying food purchasing patterns, and implementing pricing and other promotional activities to increase selection of lower-sodium foods. NYSDOH supported this work by developing the Sodium Practices Assessment Tool (SPAT) (Appendix).

SPAT uses an interview format to assess modifiable food preparation, presentation, and purchasing practices. It is based on existing tools and resources, including the Restaurant Assessment Tool and Evaluation (15), the Healthy Hospital Environment Scan (16), the Healthy Hospital Food Initiative’s pantry observation form (17), a food service company’s self-assessment checklist (18), and sodium reduction suggestions from government websites (19,20). SPAT respondents indicate how often common strategies are used at their site: never, rarely, sometimes, most of the time, always, or not applicable. They then use an action plan checklist to select strategies that they want to implement at their site. The SPAT interview covers 48 strategies, and the SPAT action plan offers 31 strategies. Altogether, SPAT takes approximately 45 minutes to complete.

## Using the Sodium Practices Assessment Tool

We conducted 19 SPAT interviews in 2014 and 2015, including all 10 hospitals and 9 of 33 senior meal sites in the 5-county catchment area. Respondents were food service managers, cooks, registered dietitians, and executive directors. After the interviews, responses were aggregated and dichotomized as true (always or most of the time) and not true (never, rarely, or sometimes). The number of responses varied. The lowest response rates were in purchasing, because those strategies were added to SPAT after 8 interviews had already been conducted.

SPAT interview results highlighted opportunities for improvement (Table 1). In the food preparation category, most sites did not make soups or sauces (n = 16) or salad dressings (n = 14) from scratch, and most prepared fish with breading (n = 11). In the food presentation category, no sites offered smaller portions as a way to reduce sodium intake, and only a few provided sodium information in a brochure (n = 3). In the food purchasing category, no sites purchased lower-sodium bread products; most sites did not purchase lower-sodium salad dressings (n = 7), poultry products (n = 7), cheeses (n = 7), cold cuts and deli meats (n = 6), or canned tomato products (n = 6).

**Table 1:** Interview and Action Plan for Sites That Completed the Sodium Practices Assessment Tool (SPAT) at Baseline, New York State, 2014–2015

Sodium Practices^a^	Sites That Completed the Interview (n = 19)			Action Plan (n = 18)^b^, Sites That Selected Strategy, No.
	Sites That Responded, No.	Statement True, No.	Statement Not True, No.	
**Preparation Methods**				
Food is prepared in a kitchen at the site	19	18	1	5
Food is prepared using recipes	19	18	1	NA
Soups/sauces/stews are made from scratch without purchased soup base	18	2	16	2
Herbs and spices are used as a salt replacement	18	10	8	6
Vegetables served are fresh or frozen (without added sauce or sodium)	19	16	3	4
Olive oil/vegetable oil/unsalted butter (instead of salted butter) is used in cooking	19	11	8	NA
Salad dressing is made from scratch without added salt	18	4	14	NA
Fish is prepared without breading	19	8	11	NA
Chicken is prepared without breading	19	13	6	NA
**Presentation Methods**				
Sodium is reduced by offering smaller portion size	17	0	17	9
Fruit or vegetable (without added salt) is included in a meal	19	15	4	4
Salad dressing is on the side	19	16	3	6
Chips and French fries are automatically included in meal^c^	12	0	12	NA
Table salt is replaced with herb salt substitute	18	1	17	NA
Sodium information is posted	17	4	13	NA
Sodium information is available in a brochure	18	3	15	NA
Pizza is available^c^	14	7	7	0
Lower-sodium deli meats are available	17	9	8	10
**Purchasing strategies**				
Lower-sodium sandwich breads are purchased	10	0	10	12
Lower-sodium rolls and bagels are purchased	10	0	10	9
Lower-sodium hamburger and hot dog buns are purchased	10	0	10	9
Lower-sodium cold cuts and deli meats are purchased	9	3	6	10
Lower-sodium cheeses are purchased	10	3	7	14
Lower-sodium poultry products are purchased	9	2	7	9
Lower-sodium canned tomato products are purchased	10	4	6	14
Lower-sodium salad dressings are purchased	8	1	7	6
Lower-sodium soup bases or gravies are purchased	8	6	2	8

Abbreviation: NA, not applicable.

a Items reflect the subset of SPAT strategies that were most applicable across assessed sites.

b Items included in the interview but not in the action plan.

c For this item, “statement not true” was the preferred answer.

Results were used to develop sodium-reduction action plans at 18 sites (Table 1). In the food preparation category, the most popular strategies selected for implementation were using herbs and spices (n = 6) and preparing food at the site (n = 5). In the food presentation category, the most popular strategies were offering lower-sodium cold cuts and deli meats (n = 10) and offering smaller portion sizes (n = 9). In the food purchasing category, the most popular strategies were purchasing lower-sodium cheeses (n = 14), tomato products (n = 14), bread (n = 12), and cold cuts and deli meats (n = 10). Overall, strategies in the food purchasing category were more likely to be selected for implementation than strategies in the preparation and presentation categories.

## Using the Sodium Practices Assessment Tool to Measure Progress

Seventeen follow-up assessments were conducted in 2016, after action plans were implemented in participating hospitals and for senior meal programs. To measure change over time, baseline ratings were averaged and compared with a matched set of follow-up ratings (never = 1, rarely = 2, sometimes = 3, most of the time = 4, and always = 5). Standard deviations were examined, and paired *t* tests were used to determine the significance of observed changes. Significance was set at *P* < .05. Two sites with missing follow-up assessments were excluded from the pre–post analyses.

Pre–post comparison results demonstrated that some sodium practices had changed over time (Table 2). In the food preparation category, we found significant improvements in the use of herbs and spices (*P* = .03) and unsalted oil or butter (*P* = .001). In the food presentation category, there were significant improvements in the inclusion of fruits and vegetables (*P* = .04), the use of herb salt substitutes (*P* = .04), and selection of lower-sodium deli meats (*P* = .03). In the food purchasing category, there were significant improvements in purchasing lower-sodium sandwich breads (*P* = .049), and cold cuts and deli meats (*P* = .02).

**Table 2:** Average Item Scores for Sites That Completed the Sodium Practices Assessment Tool (SPAT), at Baseline and at Follow-up (n = 17), New York State, 2014–2016

Sodium Practices^a^	n	Average Score at Baseline	Average Score at Follow-up	Standard Deviation	*P* Value^b^
**Preparation methods**					
Food is prepared in a kitchen at the site	17	4.82	4.88	0.56	.67
Food is prepared using recipes	17	4.82	4.88	0.43	.58
Herbs and spices are used as a salt replacement	16	3.69	4.44	1.29	.03
Vegetables served are fresh or frozen (without added sauce or sodium)	17	4.24	4.71	0.94	.06
Olive oil/vegetable oil/unsalted butter (instead of salted butter) is used in cooking	14	3.71	4.57	0.77	.001
Salad dressing is labeled “low” or “reduced” sodium	14	2.00	1.86	1.41	.71
Fish is prepared without breading	17	3.53	3.71	0.95	.46
Chicken is prepared without breading	16	3.81	3.94	0.62	.43
**Presentation methods**					
Sodium is reduced by offering smaller portion size	13	1.54	1.85	1.32	.42
Fruit or vegetable (without added salt) included in a meal	16	3.94	4.81	1.59	.04
Salad dressing is on the side	17	4.35	4.47	0.49	.33
Table salt is replaced with herb salt substitute	13	1.23	2.46	1.96	.04
Sodium information is posted	14	1.71	2.21	1.70	.29
Sodium information is available in a brochure	15	1.80	2.67	1.60	.05
Pizza is available^c^	11	3.36	3.00	1.29	.37
Lower-sodium deli meats are available	13	2.77	3.92	1.68	.03
**Purchasing strategies**					
Lower-sodium sandwich breads are purchased	9	1.44	2.78	1.73	.05
Lower-sodium rolls and bagels are purchased	8	1.13	1.75	1.60	.31
Lower-sodium hamburger and hot dog buns are purchased	8	1.13	2.00	1.64	.18
Lower-sodium cold cuts and deli meats are purchased	7	2.29	4.00	1.50	.02
Lower-sodium cheeses are purchased	9	2.78	3.89	1.97	.13
Lower-sodium poultry products are purchased	7	2.71	3.71	2.65	.36
Lower-sodium canned tomato products are purchased	9	2.67	2.89	1.48	.66
Lower-sodium salad dressings are purchased	7	2.00	1.00	1.53	.13
Lower-sodium soup bases or gravies are purchased	7	4.29	4.71	0.79	.20

a Depicted items reflect the subset of SPAT strategies that were most applicable across assessed sites.

b *P* values determined using paired-sample *t* tests.

c For this item, a reduction in the average score was considered an improvement.

Results highlight the difference between popular strategies and feasible strategies. For example, most sites were interested in purchasing and serving varied lower-sodium products, but measurable improvements were only achieved with sandwich breads and deli meats. This could indicate a need for increased availability of lower-sodium products or a need for increased emphasis on consumer education. When reductions in sodium content are achieved, SPAT results can help explain how they were accomplished.

## Improving the Sodium Practices Assessment Tool for Ongoing Use

The SPAT tool focused attention and discussion on food preparation, presentation, and purchasing strategies; it informed site-specific action plans, and it was re-administered to document changes that occurred over time. As a program tool, SPAT displayed several limitations that needed to be addressed. The response options were not clearly defined, which made it difficult to differentiate between foods that were rarely purchased and foods with lower sodium that were rarely purchased. Some items appeared twice on the tool, some were included in the interview but not in the action plan, and some were included in the action plan but not in the interview. These issues have all been resolved in an updated version of SPAT that is being field-tested in universities and early childhood education centers. Both versions of SPAT were developed to assess and improve food service. Neither was tested for validity or reliability, so SPAT’s appropriateness for research has not been established.

Current levels of sodium consumption are increasing the prevalence of cardiovascular disease in the United States and worldwide. We can reduce sodium consumption by collaborating to improve the overall food environment. Food service programs that want to reduce sodium in their meals need tools like SPAT to assess and improve their sodium practices.

## Appendix Sodium Practices Assessment Tool

This appendix is available for download as a Microsoft Word file.

## References

[1] He FJ , MacGregor GA . A comprehensive review on salt and health and current experience of worldwide salt reduction programmes. J Hum Hypertens 2009;23(6):363–84. 10.1038/jhh.2008.144 19110538

[2] Appel LJ , Frohlich ED , Hall JE , Pearson TA , Sacco RL , Seals DR , The importance of population-wide sodium reduction as a means to prevent cardiovascular disease and stroke: a call to action from the American Heart Association. Circulation 2011;123(10):1138–43. 10.1161/CIR.0b013e31820d0793 21233236

[3] Briefel RR , Johnson CL . Secular trends in dietary intake in the United States. Annu Rev Nutr 2004;24(1):401–31. 10.1146/annurev.nutr.23.011702.073349 15189126

[4] US Department of Agriculture. Sodium intake of the US population. What we eat in America. Food Surveys Research Group data brief no. 8, October 2011. http://www.ars.usda.gov/SP2UserFiles/Place/80400530/pdf/DBrief/8_sodium_intakes_0708.pdf. Accessed December 16, 2015.

[5] Wright JD , Wang CY , Kennedy-Stephenson J , Ervin RB . Dietary intake of ten key nutrients for public health, United States: 1999–2000. Advance data from vital and health statistics; no. 334. Hyattsville (MD): National Center for Health Statistics; 2003. 12743879

[6] US Department of Health and Human Services and U.S. Department of Agriculture. 2015–2020 Dietary Guidelines for Americans. 8th Edition. December 2015. http://health.gov/dietaryguidelines/2015/guidelines/. Accessed September 27, 2017.

[7] Palar K , Sturm R . Potential societal savings from reduced sodium consumption in the U.S. adult population. Am J Health Promot 2009;24(1):49–57. 10.4278/ajhp.080826-QUAN-164 19750962

[8] Bibbins-Domingo K , Chertow GM , Coxson PG , Moran AE , Lightwood JM , Pletcher MJ , Projected effect of dietary salt reductions on future cardiovascular disease. N Engl J Med 2010;362(7):590–9. 10.1056/NEJMoa0907355 20089957 PMC3066566

[9] Reducing sodium. A look at state savings in health care costs. Washington (DC): Center for Science in the Public Interest; 2015. https://cspinet.org/resource/reducing-sodium. Accessed May 21, 2017.

[10] Mattes RD , Donnelly D . Relative contributions of dietary sodium sources. J Am Coll Nutr 1991;10(4):383–93. 10.1080/07315724.1991.10718167 1910064

[11] Harnack LJ , Cogswell ME , Shikany JM , Gardner CD , Gillespie C , Loria CM , Sources of sodium in US adults from 3 geographic regions. Circulation 2017;135(19):1775–83. 10.1161/CIRCULATIONAHA.116.024446 28483828 PMC5417577

[12] Institute of Medicine. Strategies to reduce sodium intake in the United States. Washington (DC): National Academy Press; 2010. 10.3945/an.110.1002 PMC3042781 22043452

[13] Cobb LK , Appel LJ , Anderson CA . Strategies to reduce dietary sodium intake. Curr Treat Options Cardiovasc Med 2012;14(4):425–34. 10.1007/s11936-012-0182-9 22580974 PMC3612540

[14] Mugavero K , Losby JL , Gunn JP , Levings JL , Lane RI . Reducing sodium intake at the community level: the sodium reduction in communities program. Prev Chronic Dis 2012;9:E168. http://www.cdc.gov/pcd/issues/2012/pdf/12_0081.pdf 10.5888/pcd9.120081 23171670 PMC3505118

[15] Schuldt J , Levings JL , Kahn-Marshall J , Hunt G , Mugavero K , Gunn JP . Reducing sodium across the board: a pilot program in Schenectady County independent restaurants. J Public Health Manag Pract 2014;20(1, Suppl 1):S31–7. 10.1097/PHH.0b013e31829d7b7c 24322813 PMC9272046

[16] Amerson N , Nelson M , Radcliffe A , Moody C , Williams L , Miles C . Adoption of sodium reduction strategies in small and rural hospitals, Illinois, 2012. Prev Chronic Dis 2014;11:E42. http://www.cdc.gov/pcd/issues/2014/13_0261.htm 10.5888/pcd11.130261 24650620 PMC3965322

[17] Moran A , Krepp EM , Johnson Curtis C , Lederer A . An intervention to increase availability of healthy foods and beverages in New York City hospitals: the Healthy Hospital Food Initiative, 2010–2014. Prev Chronic Dis 2016;13:E77. 10.5888/pcd13.150541 27281392 PMC4900821

[18] Everyday Basics Audit, Healthy For Life^TM^, Aramark (NYSE: ARMK). http://www.aramark.com/responsibility#healthandwellness. Accessed September 27, 2017.

[19] US Department of Agriculture. Just the facts! Be salt savvy-cut back on sodium. Food and Nutrition Service; 2014 Jul 449-I. https://www.fns.usda.gov/school-meals/tools-schools-sodium. Accessed January 8, 2018.. Accessed December 16, 2015.

[20] US Department of Agriculture. Salt and sodium: 10 tips to help you cut back. Alexandria (VA): Center for Nutrition Policy and Promotion; 2015 Sep 1. http://www.choosemyplate.gov/ten-tips-salt-and-sodium. Accessed December 16, 2015.

