# Supplementary material for: Improving the Food Environment in Hospitals and Senior Meal Programs
Source: Prev Chronic Dis. 2018 Feb 15;15:E22. doi: 10.5888/pcd15.170429 (PMC5814151; doi:10.5888/pcd15.170429)
Supplement: Supplementary file 1 [file 17_0429Appendix.docx]

**Sodium Practices Assessment Tool and Action Plan** (updated 6.19.2014)

**Directions for Sodium Practices Assessment Tool**

Section A gathers background information about the site. It should be administered as a “semi-structured” interview. You can use items 1 - 16 as topic guides. You do not need to read them exactly as they are written, and you can include additional items that are appropriate for the site. You may want to write responses to this section on a separate sheet of paper. ………………………………………………………………………….………………………………………………….Page 2

Sections B, C and D gather information about sodium practices at the site, including food preparation, presentation and purchasing. These sections should be administered as “structured interviews”. Read each item exactly as it is written, and record each response by marking an ‘X’ in the appropriate box. Complete these sections before you begin working at the site **and** after you finish implementing your action plan at the site…Pages 3-5

**Directions for Sodium Practices Action Plan**

After completing all relevant assessments, meet with representatives from the site to select the sodium reduction strategies that you plan to implement. Place an ‘X’ to the left of each selected strategy on the list provided. When the assessment tool and action plan are both complete, email them to Cindy Ferrari at NYS DOH (cindy.ferrari@health.ny.gov)........................................................................ ……………………………………..……………………………….……Page 6

|  | **Completion Date** | **Interviewer(s)** | **Respondent(s)** | **Job Titles** |
| --- | --- | --- | --- | --- |
| **Assessment Tool** |  |  |  |  |
| Section A |  |  |  |  |
| Section B |  |  |  |  |
| Section C |  |  |  |  |
| Section D |  |  |  |  |
| **Action Plan** |  |  |  |  |

Date:__________ Interviewer(s): ___________________________ Respondent(s): ___________________________

**A. Background Information: “This assessment tool will help us understand how food is prepared, presented and purchased at your site with respect to sodium content. The tool has two parts which may be conducted separately or together, whichever is more convenient for you. The first part is an assessment. It should be conducted with staff members who understand the food service program at your site. The second part is an action plan. It uses what was learned during the assessment to identify appropriate sodium reduction strategies for your site. I’d like to start by gathering some background information about your site.”**

1. Briefly describe all the food service options available at this site.
2. Is a nutrient analysis conducted on foods served at your site? □All □Some □None □Don’t Know
3. Are there any nutrition-related requirements for food served? □Yes □No □Don’t Know
4. Do you contract with a food service management company? □Yes □ No ( if no, skip to Q9) □Don’t Know (if don’t know, skip to Q9)
5. What is the name of the food service company? _________________________________________________________
6. When does the current food service contract start and end? Start Date_______ End Date_______
7. When will discussions about the next food service contract cycle begin? Month_______ Year_______
8. Who could I contact at this site to get more information about the food service contract? _______________________________________________
9. Approximately how many meals are served at this site on a typical day during the week (M-F) ? # __________
10. Approximately how many meals are served at this site on a typical day during the weekend (Sat-Sun) ? # __________
11. Is there a standard rotating menu at this site? □Yes □No □Don’t Know (if yes, what is the length of the cycle? _____________ )
12. What are the most popular food items (for example: hot main courses, cold sandwiches, grilled items, salads, pizza, soups, snacks, etc.)?

“**To evaluate the effectiveness of our sodium reduction activities, we would like to track some information about food items over time.”**

1. Would it be possible for us to obtain nutrient information on selected items for this purpose? □Yes □No □Don’t Know
2. Would it be possible for us to obtain production information on selected items for this purpose? □Yes □No □Don’t Know
3. Would it be possible for us to obtain sales information on selected items for this purpose? □Yes □No □Don’t Know
4. Who should I contact to obtain this information? _________________________________________________________

Date:__________ Interviewer(s): ___________________________ Respondent(s): ___________________________

1. **Food Preparation: “Now I am going to read a list of food preparation methods. Please indicate how often each one is true at your site. You can say: never, rarely, sometimes, most of the time, always…or you can say ‘not applicable’. There is also a space for comments if you need to clarify an answer. ”**

| **B** | **Preparation Methods** | Never | Rarely | Some-times | Most of the time | Always | N/A | Comments |
| --- | --- | --- | --- | --- | --- | --- | --- | --- |
| 1 | Food is prepared in a kitchen at the site |  |  |  |  |  |  |  |
| 2 | Food is prepared using recipes |  |  |  |  |  |  |  |
| 3 | Salt is measured when cooking |  |  |  |  |  |  |  |
| 4 | Salt is added to cooking water for pasta, vegetables, etc. (*reverse scale*) |  |  |  |  |  |  |  |
| 5 | Soups/sauces/stews are made from scratch without purchased soup base |  |  |  |  |  |  |  |
| 6 | Reduced sodium soup base is used (defined as at least less than 25% of original product) |  |  |  |  |  |  |  |
| 7 | High sodium soup base is diluted |  |  |  |  |  |  |  |
| 8 | Higher sodium soup base is used as a flavoring ingredient in dishes other than soups (*reverse scale*) |  |  |  |  |  |  |  |
| 9 | Herbs and spices are used as a salt replacement |  |  |  |  |  |  |  |
| 10 | Vegetables served are fresh or frozen (without added sauce or sodium) |  |  |  |  |  |  |  |
| 11 | Olive oil/vegetable oil/unsalted butter is used in cooking (instead of salted butter) |  |  |  |  |  |  |  |
| 12 | French fries have no added salt and are not coated |  |  |  |  |  |  |  |
| 13 | Salad dressing is made from scratch without added salt |  |  |  |  |  |  |  |
| 14 | Salad dressing is labeled “low” or “reduced” sodium |  |  |  |  |  |  |  |
| 15 | Fish is prepared without breading |  |  |  |  |  |  |  |
| 16 | Chicken is prepared without breading |  |  |  |  |  |  |  |

**“Thank you for answering these questions about food preparation methods at your site.”**Date:__________ Interviewer(s): ___________________________ Respondent(s): ___________________________

1. **Food Presentation: “Now I am going to read a list of food presentation methods. Please indicate how often each one is true at your site. You can say: never, rarely, sometimes, most of the time, always…or you can say ‘not applicable’. There is also a space for comments if you need to clarify an answer. ”**

| **C** | **Presentation Methods** | Never | Rarely | Some-times | Most of the time | Always | N/A | Comments |
| --- | --- | --- | --- | --- | --- | --- | --- | --- |
| 1 | Salt reduced by offering smaller portion size |  |  |  |  |  |  |  |
| 2 | Fruit or vegetable (without added salt) included in a meal |  |  |  |  |  |  |  |
| 3 | Substitute fruit or vegetable (without added salt) for fries or chips at no additional cost |  |  |  |  |  |  |  |
| 4 | Salad dressing is on the side |  |  |  |  |  |  |  |
| 5 | Adding cheese or bacon to sandwiches costs more |  |  |  |  |  |  |  |
| 6 | Chips and fries are automatically included in meal (*reverse scale*) |  |  |  |  |  |  |  |
| 7 | Table salt is replaced with herb salt substitute |  |  |  |  |  |  |  |
| 8 | Sodium information is posted |  |  |  |  |  |  |  |
| 9 | Sodium information is available in a brochure |  |  |  |  |  |  |  |
| 10 | Lower sodium options are placed near point-of-purchase |  |  |  |  |  |  |  |
| 11 | Snack food is packaged in single serving sizes |  |  |  |  |  |  |  |
| 12 | A la carte pretzels and chips are placed near point-of-purchase (*reverse scale*) |  |  |  |  |  |  |  |
| 13 | Lower sodium vegetable juice is available |  |  |  |  |  |  |  |
| 14 | Salad bar includes high sodium items like olives, pickles, beets, canned beans , etc. (*reverse scale*) |  |  |  |  |  |  |  |
| 15 | Pizza is available (*reverse scale*) |  |  |  |  |  |  |  |
| 16 | Lower sodium deli meats are available |  |  |  |  |  |  |  |

**“Thank you for answering these questions about food presentation methods at your site.”**

Date:__________ Interviewer(s): ___________________________ Respondent(s): ___________________________

**D. Food Purchasing: “Now I am going to read a list of food purchasing strategies. Please indicate how often each one is true at your site. You can say: never, rarely, sometimes, most of the time, always…or you can say ‘not applicable’. There is also a space for comments if you need to clarify an answer. ”**

| **D** | **Food Purchasing Strategies** | Never | Rarely | Some-times | Most of the time | Always | N/A | Comments |
| --- | --- | --- | --- | --- | --- | --- | --- | --- |
| 1 | Lower sodium sandwich breads are purchased |  |  |  |  |  |  |  |
| 2 | Lower sodium rolls and bagels are purchased |  |  |  |  |  |  |  |
| 3 | Lower sodium hamburger and hot dog buns are purchased |  |  |  |  |  |  |  |
| 4 | Lower sodium hot dogs are purchased |  |  |  |  |  |  |  |
| 5 | Lower sodium cold cuts and deli meats are purchased |  |  |  |  |  |  |  |
| 6 | Lower sodium cheeses are purchased |  |  |  |  |  |  |  |
| 7 | Lower sodium bacon and/or sausage is purchased |  |  |  |  |  |  |  |
| 8 | Lower sodium poultry products are purchased |  |  |  |  |  |  |  |
| 9 | Lower sodium canned tomato products are purchased |  |  |  |  |  |  |  |
| 10 | Lower sodium canned soups are purchased |  |  |  |  |  |  |  |
| 11 | Lower sodium canned vegetables are purchased |  |  |  |  |  |  |  |
| 12 | Lower sodium pizzas are purchased |  |  |  |  |  |  |  |
| 13 | Lower sodium chips, popcorn or pretzels are purchased |  |  |  |  |  |  |  |
| 14 | Lower sodium salad dressings are purchased |  |  |  |  |  |  |  |
| 15 | Lower sodium soup bases or gravies are purchased |  |  |  |  |  |  |  |
| 16 | Other lower sodium products are purchased (please specify in comments) |  |  |  |  |  |  |  |

**“Thank you for answering these questions about food purchasing strategies at your site.”**

Date:__________ Interviewer(s): __________ ____________ Respondent(s): ______________________

**Sodium Practices Action Plan**

| **B** | **Food Preparation Strategies** |
| --- | --- |
|  | Gradually reduce added salt in recipes and replace with herbs or salt substitute |
|  | Do not add salt when cooking or boiling water |
|  | Use half water/half soup base to reduce salt in soups, stews, marinades and gravies |
|  | Make soup stock from scratch and eliminate soup base |
|  | Replace canned vegetables with lower sodium frozen or fresh vegetables |
|  | Adjust menu cycle to reduce the number of meals that rely on processed or prepared foods |
|  | Other preparation strategies (specify): |
| **C** | **Food Presentation Strategies** |
|  | Reduce portion sizes for high sodium foods |
|  | Include fresh fruit and vegetable sides |
|  | Substitute fruits, vegetables or lower sodium options in place of chips or fries at no extra cost |
|  | Offer salad dressing in pre-measured (max 2 ounce) containers on the side |
|  | Prepare sandwiches without cheese or bacon |
|  | Identify and promote benefits of lower sodium options |
|  | Offer taste tests of lower sodium menu changes |
|  | Remove pretzels and chips as an a la carte option |
|  | Reduce the number of times per week that pizza is served |
|  | Offer lower sodium deli meats |
|  | Other presentation strategies (specify): |
| **D** | **Food Purchasing Strategies** |
|  | Identify and purchase lower sodium sandwich breads |
|  | Identify and purchase lower sodium rolls and bagels |
|  | Identify and purchase lower sodium hamburger and hot dog buns |
|  | Identify and purchase lower sodium hamburgers and hot dogs |
|  | Identify and purchase lower sodium cold cuts and deli meats |
|  | Identify and purchase lower sodium cheeses |
|  | Identify and purchase lower sodium bacon and/or sausage |
|  | Identify and purchase lower sodium poultry products |
|  | Identify and purchase lower sodium canned tomato products |
|  | Identify and purchase lower sodium canned soup |
|  | Identify and purchase other lower sodium canned vegetables |
|  | Identify and purchase lower sodium pizzas |
|  | Identify and purchase lower sodium chips, popcorn, and pretzels |
|  | Identify and purchase lower sodium salad dressings |
|  | Identify and purchase lower sodium soup bases and gravies |
|  | Other purchasing strategies (specify): |
